# Improved whole-brain reconfiguration efficiency reveals mechanisms of speech rehabilitation in cleft lip and palate patients: an fMRI study

**DOI:** 10.3389/fnagi.2025.1536658

**Published:** 2025-03-04

**Authors:** Wenjing Zhang, Qian Si, Zhongtian Guan, Lei Cao, Mengyue Wang, Cui Zhao, Liwei Sun, Xu Zhang, Zhixi Zhang, Chunlin Li, Weiqun Song

**Affiliations:** ^1^Department of Rehabilitation Medicine, Xuanwu Hospital Capital Medical University, Beijing, China; ^2^School of Cyber Science and Technology, Beihang University, Beijing, China; ^3^School of Biomedical Engineering, Capital Medical University, Beijing, China; ^4^School of Life Sciences, Beijing Institute of Technology, Beijing, China; ^5^School of Communication Sciences, Beijing Language and Culture University, Beijing, China; ^6^Aerospace Information Research Institute, Chinese Academy of Sciences, Beijing, China

**Keywords:** CLP, speech rehabilitation, functional connectivity, fMRI, reconfiguration efficiency, biomarker

## Abstract

**Introduction:**

Cleft lip and/or palate (CLP) patients still have severe speech disorder requiring speech rehabilitation after surgical repair. The clarity of language rehabilitation is evaluated clinically by the Language Rehabilitation Scale. However, the pattern and underlying mechanisms of functional changes in the brain are not yet clear. Recent studies suggest that the brain’s reconfiguration efficiency appears to be a key feature of its network dynamics and general cognitive abilities. In this study, we compared the association between rehabilitation effects and reconfiguration efficiency.

**Methods:**

We evaluated CLP patients with speech rehabilitation (*n* = 23) and without speech rehabilitation (*n* = 23) and normal controls (*n* = 25). Assessed CLP patients on the Chinese Speech Intelligibility Test Word Lists and collected fMRI data and behavioral data for all participants. We compared behavioral data and task activation levels between participants for between-group differences and calculated reconfiguration efficiencies for each task based on each participant. In patients, we correlated reconfiguration efficiency with task performance and measured the correlation between them.

**Results:**

Behaviorally, CLP patients with rehabilitation scored significantly higher than those without rehabilitation on the Chinese Speech Intelligibility Test Word Lists. Rehabilitation caused local brain activation levels of CLP patients to converge toward those of controls, indicating rehabilitative effects on brain function. Analysis of reconfiguration efficiency across tasks at the local and whole-brain levels identified underlying recovery mechanisms. Whole-brain reconfiguration efficiency was significantly and positively correlated with task performance.

**Conclusion:**

Our results suggest that speech rehabilitation can improve the level of language-related brain activity in CLP patients, and that reconfiguration efficiency can be used as an assessment index of language clarity to evaluate the effectiveness of brain rehabilitation in CLP patients, a finding that can provide a better understanding of the degree of brain function recovery in patients.

## 1 Introduction

Cleft lip and/or palate (CLP) is the most frequent congenital defect that occurs in the area of the face (Mangold et al., 2017; Antoszewski and Fijałkowska, 2018). CLP is estimated to occur in 1 in every 700 newborns (Crockett and Goudy, 2014; Gatti et al., 2017). Currently, fissure repair surgery is mainly used to treat CLP patients (Becker et al., 2008; Alois and Ruotolo, 2020). However, even after corrective surgery, children with CLP will continue to struggle with speech and language difficulties, which can affect them for the rest of their lives (Alois and Ruotolo, 2020). Studies have shown that speech rehabilitation for dysarthria can improve the speech clarity of patients (Lin et al., 2014). Speech intelligibility is an indispensable metric for determining the effectiveness of rehabilitation, but currently, there is no widely used standardized intelligibility test. In clinical practice, therapists assess the effectiveness of speech rehabilitation by scoring the patient’s Chinese Speech Intelligibility Test Word Lists (CSITWL), which results in the apparent effectiveness of the speech rehabilitation of the patient being greatly influenced by the subjective evaluation factors of the therapists (Ribeiro et al., 2021). In addition, the patterns of brain function change and the underlying mechanism are unclear.

Functional magnetic resonance imaging (fMRI) is a noninvasive brain imaging technique that has been widely used in recent years to study the functional activity of the brain (Heeger and Ress, 2002; Logothetis, 2008; Craddock et al., 2015). Studies have shown that the structure and function of the brains of CLP patients are significantly different from those of normal people. Tollefson and Sykes documented Ellen et al’s findings that CLP may be associated with structural brain abnormalities, and these findings highlight the important relationship and interactions between CLP and brain development (Nopoulos et al., 2002a; Tollefson and Sykes, 2010). Another study showed that brain activation in CLP patients differed significantly from that in normal individuals, showing specific intellectual and cognitive deficits when performing language tasks (Nopoulos et al., 2002b; Sato-Wakabayashi et al., 2008). Some of researches compared the brain structure and functional changes among CLP adults with speech disorders, CLP individuals who underwent speech rehabilitation training, and normal controls. The results indicated that the brain function and structure of CLP individuals who have received speech rehabilitation training tend to approximate normal controls more closely in terms of their brain structures, activation patterns, and functional connectivity (FC) networks. More importantly, there are notable differences compared with CLP with cleft palate-related speech disorder. This reflects that speech rehabilitation training can partially improve the CLP patient brain function recovery and help their brains function gradually toward normal. In this way, it provides neural mechanism evidence for partial effectiveness of the speech rehabilitation treatments (Li et al., 2020; Sun et al., 2022). None of these studies evaluated the effects of rehabilitation on brain mechanisms in CLP patients after surgery. Therefore, we needed to investigate the brain mechanisms underlying rehabilitation in CLP patients.

Many theories of brain function rely on the fact that cooperative and effective communication between spatially separated neural regions is essential for the execution of effective behavior (Chang and Glover, 2010; Fedorenko and Thompson-Schill, 2014). That is, no one region works in isolation. To fully understand the utility of a particular region of the brain, we must consider both local and global structures that nourish and constrain ongoing activity during behavior (Cole et al., 2013b; Braun et al., 2015). FC has become an important aspect of many studies, especially fMRI studies (Cohen et al., 2008; Cole et al., 2021). In recent studies, FC has been used to differentiate groups of participants and to explain how tasks reconfigure brain networks. In all of these approaches, the concept of similarity in brain network FC has been used (Van Den Heuvel et al., 2009; Cole et al., 2013a; Venkatesh et al., 2020). A study on FC showed that the efficiency of updating one’s brain from the resting state to a task-based state is positively correlated with an individual’s ability to perform a wide range of cognitively challenging tasks (Poldrack, 2015; Schultz and Cole, 2016). Brain network structures in the resting state have become more closely aligned with an individual’s network structure during various tasks, and the brain’s reconfiguration efficiency when task demands change is a marker of high intelligence (Hearne et al., 2017). Consequently, reconfiguration efficiency is a valid means of evaluating brain cognitive function.

Recent studies have shown that changes in task-related functional network organization are small relative to changes in the overall functional network organization during resting states and particular tasks, suggesting that changes in task-related cognition correspond to small functional network changes (Cole et al., 2014). Most CLP patients maintain the same minimal cognitive abilities as healthy individuals, and based on this, we hypothesized that the overall intrinsic functional network structure supports these cognitive abilities through specific network organization, which would be similar between patients and controls and that rehabilitation would alter only a small proportion of the associated functional networks with a high degree of specificity.

Here, we used Pearson’s correlation coefficients to represent the brain’s reconfiguration efficiency while performing the task (Betzel et al., 2016). We quantified all FC changes at once from the perspective of the entire connectome to provide information about the overall pattern of the intrinsic network structure. Additionally, task-based FC between locally activated brain regions was calculated, and the reconfiguration efficiency between task-related functional brain networks was measured. We hypothesized that with rehabilitation, the brain structure and function of CLP patients would tend to be more normal; in other words, their reconfiguration efficiency would increase and approach that of normal people (Cole et al., 2019). We used these methods to compare the ability to complete tasks among CLP patients with and without rehabilitation and normal controls, which provided a new way to measure changes in brain function during rehabilitation. Testing hypotheses regarding brain intrinsic architecture pattern similarity across reading and subvocalization tasks would enable us to put CLP-related FC alterations in perspective and possibly help to explore whether reconfiguration efficiency can be used as a biomarker to evaluate the effects of rehabilitation for CLP patients.

## 2 Materials and methods

### 2.1 Participants

A total of 71 participants were recruited in this experiment. Participants were recruited from Beijing Stomatology Hospital. All participants signed informed consent. Participants had normal or corrected eyesight, and they were all right-handed. The procedures used in this study were in accordance with the World Medical Association’s “Code of Ethics” (Declaration of Helsinki) and the standards established by the Institutional Ethics Review Board of Capital Medical University, China. The participants were divided into three groups. The after-speech rehabilitation CLP patients (Aclp): This group, Aclp, consisted of 23 nonsyndromic CLP (NSCLP) patients who started speech rehabilitation 6 months after undergoing corrective surgery for CLP. The patients had received treatment once daily for 30 min in each session and for 30 days. And they also had scored above 86 points out of 100 points on the CSITWL. The before speech rehabilitation CLP patients (Bclp): 23 NSCLP patients without speech rehabilitation. The normal controls (Norm): 25 normal participants. The specific demographic and clinical data for each group of participants are shown in Table 1. There were no significant differences in age, sex, or years of education among the three groups.

**TABLE 1:** Demographic and clinical data of the participants.

	Number of participants	Age	Sex (M/F)	Years of education	Speech score
Aclp	23	23.86 ± 4.98	14/9	13.80 ± 2.59	93.6 ± 5.00
Bclp	23	24.39 ± 5.61	11/12	13.48 ± 2.25	49.82 ± 23.62
Norm	25	23.08 ± 4.01	16/9	14.76 ± 1.85	–
*p*	–	0.635^a^	0.492^b^	0.124^a^	<10^–6^ ^c^

*^a^*One-way analysis of variance.

*^b^*Chi-square test.

*^c^*Independent sample *t*-test.

### 2.2 Experimental paradigm

Each participant completed one resting-state MRI session and two task-based MRI sessions. Regarding the imaging sessions, the participants first received a resting-state scan, then two task-based scans, and finally a structural scan.

**FIGURE 1:**
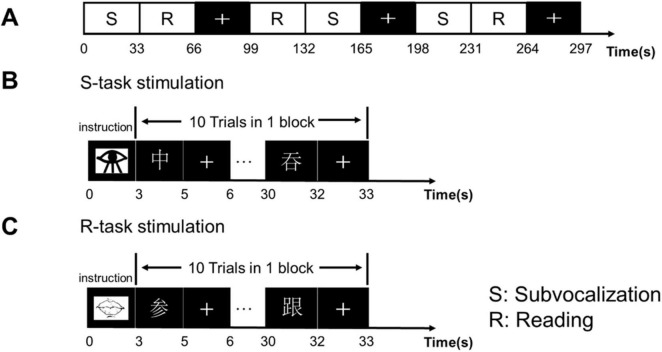
Experimental design and sequence. **(A)** The experimental design used a block design with a task block and resting block that alternated three times. Each block lasted 33 s. In the resting block, the “+” sign appeared for 33 s, requiring the participants to look at the “+” in the center of the screen and to keep the head stationary. **(B)** Subvocalization task: started with the appearance of a picture of an eye that lasted 3 s, and was followed by alternating presentations of a Chinese character and “+” that repeated 10 times. The participants were asked to press the response key after mentally reading the character as clearly as possible. **(C)** Reading task: started with the appearance of a picture of lips, followed by Chinese characters and “+,” repeated 10 times. The participants were required to read the Chinese characters as clearly as possible and then pressed the response key as soon as possible.

In the task-based scans, the participants were required to complete two different tasks, a reading task and a subvocalization task. The reading task was specific to the speech rehabilitation received by the CLP patients. Fluency in reading was related to phonological awareness and visual memory, and the participants were asked to look at the Chinese characters on the projection screen through a mirror, read the characters as clearly as possible, and then press the response key as soon as possible. The subvocalization task required only that the participants mentally read without making a sound and press the response key (Figure 1).

### 2.3 Data collection

Imaging data were collected using a 3T Siemens MR scanner fitted with a 32-channel head coil. The resting-state and task-based data collection parameters were as follows: 32 slices were acquired every 2,000 ms (field of view, 240 mm; echo time, 50 ms; flip angle, 90°; voxel dimensions, 3 mm^3^). The structural images were collected for preprocessing functional image registration. The acquisition parameters were as follows: magnetization-prepared two rapid acquisition gradient echoes sequence (slices, 192; field of view, 240 mm; echo time, 50 ms; flip angle, 10°; voxel dimensions, 1 mm^3^). Behavioral data were collected from participants during the fMRI scans, recording their accuracy in each task as well as their median response time. CSITWL were conducted with CLP patients.

### 2.4 Data preprocessing

We used an adapted version of the MATLAB (MathWorks 2018) toolbox data processing software to preprocess the imaging data (SPM 12). Both resting-state and task-based data were preprocessed using the same pipeline. T1 images were reoriented, skulls were stripped, and NIfTI functional images were collated using statistical parameter mapping (Spronk et al., 2021). For each gray matter voxel, we regressed the following signals: undesired linear trend and signals from six head motion parameters, cerebrospinal fluid, and white matter (Glasser et al., 2013). To regress the residual signals unrelated to the neural activity, we used the CompCor method (Behzadi et al., 2007). Because the global signal regression step is currently controversial, we did not perform regression on the global signal (Li et al., 2019). This choice may result in more motion artifacts in the data. For this reason, we used a rigorous head movement review approach (Van Dijk et al., 2012). The participants with a shift in head position greater than 3 mm in any one scan were excluded. Frame-to-frame displacements greater than 0.40 mm were rejected. The single-object functional image was subsequently normalized and smoothed (FWHM = 4 mm).

### 2.5 Task activation level estimation

Task activation magnitude was estimated using a standard general linear model (GLM). Familywise error (FWE) was used to correct for multiple comparisons.

### 2.6 Selection of ROIs

To remove pseudo-activation, we used the comparison images from the Norm group as a mask (*p* < 0.05). Subsequently, we performed a one-way analysis of variance (ANOVA) on the comparison images of the three groups of participants under the reading task and the subvocalization task to obtain the results of activated brain areas that differed between the three groups of participants under the two tasks (Craddock et al., 2012). The ROIs for the task-based fMRI were selected by intersecting the differentially activated brain regions (*F* > 11.46, *p* < 0.05, FWE cluster-level corrected) with a sphere with a radius 5 or 8 mm and were centered on the voxel with the largest local *F* (*F* > 14.04, *p* < 0.05, FWE cluster-level corrected).

### 2.7 Selection of brain network templates

We used two types of brain templates to explore regional- and system-level issues. The anatomical automatic labeling 1024 (AAL-1024) atlas was generated from the AAL-116 atlas and could provide 1,024 partitions of the same area to avoid the additional effect of different partition areas. More features could be extracted from the FC, with more reliable and effective predictions for subtle changes in the whole-brain network when the brain is performing the task. Using the ROIs obtained based on task-activated brain regions, we were able to build a regional brain network template for that task. This network provided information about the connections between brain regions closely related to the task.

### 2.8 Reconfiguration efficiency

Average time series of all voxels in fMRI extracted using selected brain templates. By using the residuals from the standard GLM regression of task events, we eliminated the average task-related signal from the task data (Cole et al., 2014). This approach has been shown to improve the reliability of task FC estimation (Power et al., 2014). We concentrated on the similarity in FC patterns as a measure of functional network updates. In this study, Pearson’s correlation coefficient was used as an efficiency measure (Cao et al., 2014). We calculated Pearson correlations between paired brain regions for each subject and each task. Normalization of Pearson’s correlation values was performed using Fisher’s *Z* transformation. These normalized values were then used for all subsequent statistical tests. It was important to note that using Fisher’s *Z* transformation limited our use of similarity values. We then calculated the similarity between each participant’s resting-state FC and each task-based FC separately as the reconfiguration efficiency of that task. We considered only the upper triangle of the FC matrix. Due to the number of statistical tests, we report the false discovery rate (FDR)-corrected *p* values for the preliminary analysis.

### 2.9 Calculated the correlation between reconfiguration efficiency and task performance

**FIGURE 2:**
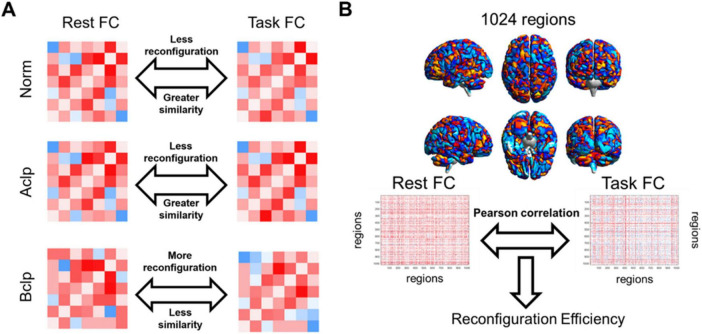
Rationale for evaluating the efficiency of FC network architecture reconfiguration. **(A)** We hypothesized that CLP patients with speech rehabilitation in the Aclp group had a higher similarity between resting-state FC and task-based FC, had a more efficient FC update efficiency, and were closer to normal participants than CLP patients without speech rehabilitation in the Bclp group. This conceptual diagram illustrated our assumptions about the reconfiguration efficiency in different groups. **(B)** In this study, mean time series were extracted from the AAL-1024 atlas, and paired correlations were calculated between each participant’s resting and task states. The reconfiguration efficiency was then computed by calculating the similarity between the task-based FC and the resting-state FC.

We used CSITWL scale scores to represent participants’ task performance. We then calculated Pearson’s correlations between reconfiguration efficiency and performance on the reading task for CLP patients based on different brain templates (FDR was corrected for multiple comparisons) (Figure 2).

## 3 Results

### 3.1 Behavioral characteristics

We performed a statistical analysis of the keystroke response time during the reading task. The median reaction time was as follows: Aclp group: 1.11 ± 0.38 s; Bclp group: 1.10 ± 0.39 s; and Norm group: 0.83 ± 0.28 s. A two-by-two comparison showed significant differences in median response times between the Aclp and Bclp groups (*p* < 0.001, *t* = 3.26), the Aclp and Norm groups (*p* < 1 × 10^–6^, *t* = 7.75), and the Bclp and Norm groups (*p* < 0.004, *t* = 2.89). These results suggested that the normal participants completed the task the fastest, followed by the patients in Bclp group, and the patients in Aclp group responded the slowest. We assessed the accuracy of the participants’ key presses. Results showed that the participants’ accuracy rates were all close to 100%, with no statistically significant differences.

The median response time in the subvocalization task was as follows: Aclp group: 1.11 ± 0.38 s; Bclp group: 1.10 ± 0.39 s; and Norm group: 0.83 ± 0.28 s. There were statistically significant differences in the median reaction times between the Aclp and Norm groups (*p* < 1 × 10^–4^, *t* = 9.07) and the Bclp and Norm groups (*p* < 1 × 10^–6^, *t* = 7.99). The results showed that the Norm group had the shortest response time, and the CLP patients had longer response times. The participants’ keystroke accuracy rates were close to 100%, with no statistically significant differences.

### 3.2 Differences in local brain area activation between groups

**FIGURE 3:**
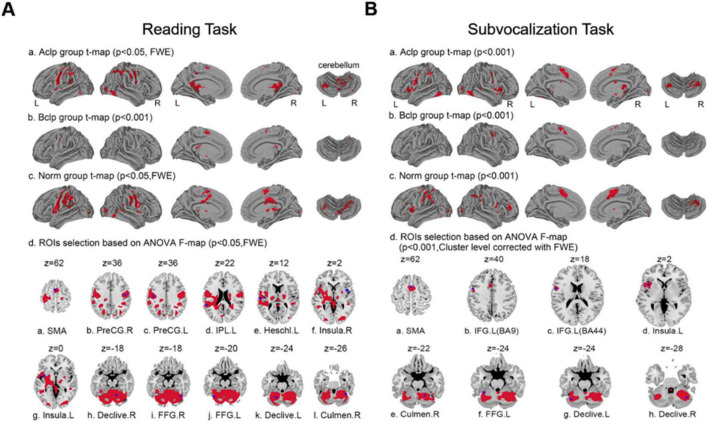
Group-level activation results and selection of ROIs for the three groups of participants based on performance in different tasks. **(A)** Plots a, b, and c show the group-level activation results in the three groups of participants during the reading task; plot d shows the 12 ROIs selected by an *F* test of differentially activated brain regions. **(B)** Plots a, b, and c show the group-level activation results in the three groups of participants during the subvocalization task; plot d shows the eight ROIs selected by an *F* test of differentially activated brain regions.

We visualized group-level activation results from the three groups of participants engaged in a reading task and a subvocalization task (Figure 3). During the reading task, the brain regions significantly activated in the Aclp group (*p* < 0.05, FWE corrected) included the cerebellum, cingulate gyrus, precentral gyrus, and parahippocampal gyrus. There was significant activation (*p* < 0.001) in brain regions that included the caudate nucleus, cingulate gyrus, and middle frontal gyrus in the Bclp group. In the Norm group (*p* < 0.05, FWE corrected), brain areas that were significantly activated included the precentral gyrus, cerebellum, postcentral gyrus, middle frontal gyrus, Broca’s area, and Wernicke’s area. During the subvocalization task, brain areas significantly activated in the Aclp group (*p* < 0.001) included the cerebellum, frontal middle gyrus, and insula; the Bclp group (*p* < 0.001) included the precentral gyrus, middle frontal gyrus, and superior frontal gyrus; and the Norm group (*p* < 0.001) included the cerebellum, precentral gyrus, and middle frontal gyrus.

We selected ROIs with an *F* test of the differentially activated brain regions; in the reading task, 12 ROIs were selected based on the differential activation results of the *F* test (*p* < 0.05, FWE corrected) across the three groups of participants. Table 2 shows the specific names of these ROIs, their abbreviations and their coordinates. Similarly, eight ROIs were selected for the subvocalization task. The specific information is shown in Table 3.

**TABLE 2:** Center coordinates of ROIs in the reading task.

Sort	Abbreviation	Anatomical area	*F*	*X*	*Y*	*Z*	*r*
1	SMA	Auxiliary exercise area of upper forehead	27.41	0	4	62	5
2	PreCG.R	Right central anterior gyrus	54.89	46	−12	36	8
3	PreCG.L	Left central anterior gyrus	49.34	−42	−14	36	5
4	IPL.L	Left apical and inferior lobule	15.51	−58	−42	22	8
5	Heschl.L	Left lateral temporal gyrus	22.20	−62	−18	12	5
6	Insula.R	Right insula	12.28	42	4	2	5
7	Insula.R	Left insula	16.37	−40	4	0	5
8	Declive.R	Right cerebellar hillside	33.64	14	−60	−18	8
9	FFG.R	Right spindle gyrus	14.04	44	−58	−18	5
10	FFG.L	Left spindle gyrus	15.90	−40	−64	−20	5
11	Declive.L	Left cerebellar hillside	27.85	−20	−60	−24	5
12	Culmen.R	Right cerebellar hilltop	22.95	38	−50	−26	5

**TABLE 3:** Center coordinates of ROIs in the subvocalization task.

Sort	Abbreviation	Anatomical area	*F*	*X*	*Y*	*Z*	*r*
1	SMA	Auxiliary exercise area of upper forehead	24.40	0	4	62	8
2	IFG.L(BA9)	Left lower forehead back BA9	8.41	−50	4	40	8
3	IFG.L(BA44)	Left lower forehead back BA44 Broca	10.38	−56	4	18	5
4	Insula.L	Left insula BA13	8.53	−42	14	2	5
5	Culmen.R	Right cerebellar hilltop	16.55	16	−52	−22	8
6	FFG.L	Left spindle gyrus BA37	11.49	−38	−46	−24	8
7	Declive.L	Left cerebellar hillside	10.85	−38	−64	−24	5
8	Declive.R	Right cerebellar hilltop	14.34	40	−62	−28	8

**FIGURE 4:**
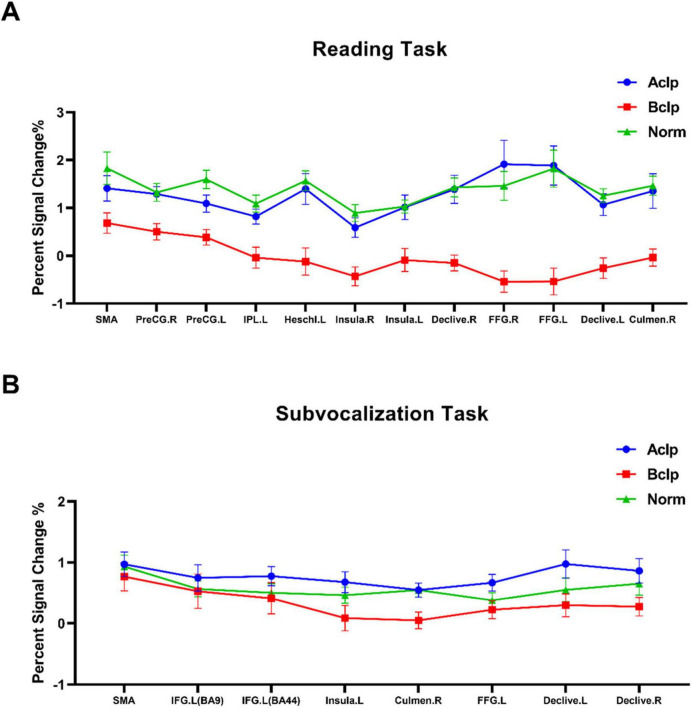
Percent signal change in ROIs for the three groups of participants. **(A)** The percent signal change observed in the participants during the reading task for 12 ROIs based on *F* tests of differentially activated brain regions. **(B)** Percent signal change observed in participants during the subvocalization task for eight ROIs based on *F* tests of differentially activated brain regions. Error lines in the figure represent the standard error of the mean (SEM).

Percent signal change is a more intuitive concept than parametric weighting and evaluating a raw percent signal change is closer to the data than evaluating a statistical metric filtered through multiple layers of temporal preprocessing and statistical evaluation. Based on the ROIs selected by the *F* test for differentially activated brain regions in the different tasks, we visualized brain signal changes at each ROI for the three groups of subjects (Figure 4).

### 3.3 Significant improvement in brain reconfiguration efficiency in CLP patients with speech rehabilitation

**FIGURE 5:**
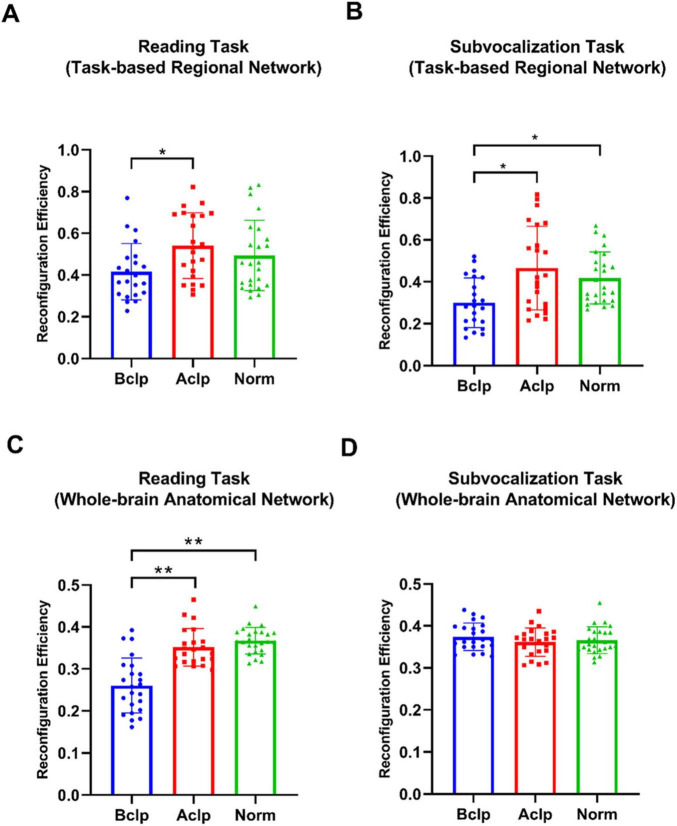
Reconfiguration efficiency in the three groups of participants in different tasks. **(A)** Reconfiguration efficiency in three groups in the reading task-activated brain network, and there are significant differences only between the Bclp and the Aclp group (*p* < 0.05). **(B)** Reconfiguration efficiency in the three groups in the subvocalization task-activated brain network, and the results show significant differences between the Bclp and the Aclp group and between the Bclp and the Norm group (*p* < 0.05). **(C)** Reconfiguration efficiency in the three groups in the reading task based on AAL-1024 atlas, and the results show that the reconfiguration efficiency in the Bclp and Aclp group and in the Bclp and Norm group were significantly different (*p* < 0.01), with no significant difference between the Aclp and Norm groups. **(D)** Reconfiguration efficiency in the three groups in the subvocalization task based on AAL-1024 atlas, and the results show that there were no significant differences in reconfiguration efficiency among the three groups. Error lines in the figure represent standard deviations (SDs). *p < 0.05; **p < 0.01.

Based on the ROIs selected from the three groups of participants based on differential activation of local brain regions during the reading task and the subvocalization task, we constructed regional networks for the 12 ROIs activated by the reading task and the 8 ROIs activated by the subvocalization task. FC reconfiguration efficiency was calculated by comparing the regional network FC configuration in each task with the FC configuration during the resting state. Similarly, the AAL-1024 whole-brain atlas was used with standard Pearson’s correlation-based FC measurements, and we calculated the FC weights for each connection in the 1,024 × 1,024 matrix. The mean time series from the resting-state fMRI and task-based fMRI data were extracted in the same way, and the reconfiguration efficiency for the whole brain was calculated. We used two-tailed, paired *t* tests to statistically analyze the differences in reconfiguration efficiency between groups (we used “*r*” to denote reconfiguration efficiency). When using a reading task-based regional network atlas, we found significant differences only between the Bclp group and the Aclp group (*p* = 0.0068), with the Aclp group having higher reconfiguration efficiency (Bclp: *r* = 0.42, Aclp: *r* = 0.54, and Norm: *r* = 0.49). When using a subvocalization task-based functional network atlas, we found significant differences between the Bclp group and the Aclp group (*p* = 0.0018) and between the Bclp group and the Norm group (*p* = 0.0018), with the Aclp group having a higher reconfiguration efficiency than the Bclp group that was closer to that in the Norm group (Bclp: *r* = 0.30, Aclp: *r* = 0.46, and Norm: *r* = 0.42). When using the whole-brain anatomical network AAL-1024 atlas, we found that there were significant differences between the Bclp and Aclp groups (*p* < 0.0001), and between the Bclp and Norm groups (*p* < 0.0001) in the reading task: reconfiguration efficiency is significantly higher in CLP patients with speech rehabilitation (Bclp: *r* = 0.26, Aclp: *r* = 0.35, and Norm: *r* = 0.36). The reconfiguration efficiency in the three groups of participants in the subvocalization task, on the other hand, was not significantly different (Bclp: *r* = 0.37, Aclp: *r* = 0.36, and Norm: *r* = 0.37) (Figure 5).

### 3.4 Higher brain reconfiguration efficiency is associated with better task performance

**FIGURE 6:**
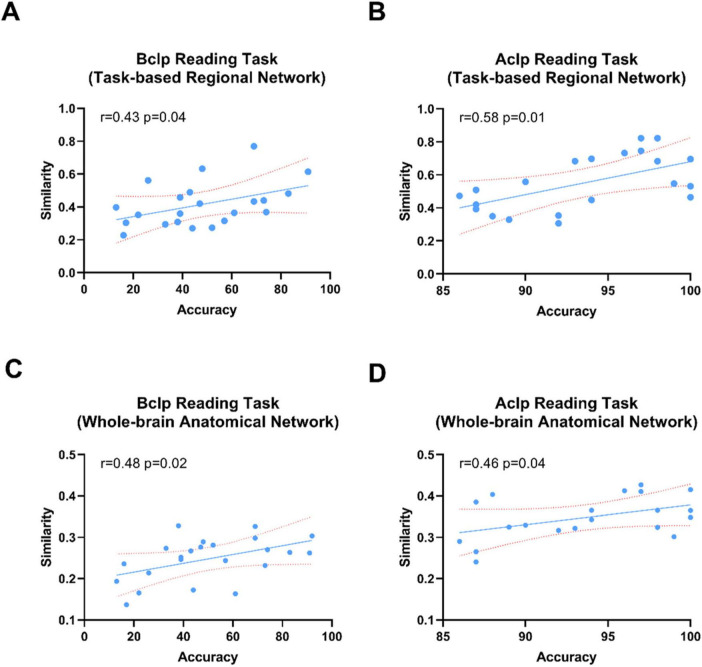
Correlations between patients’ reconfiguration efficiency based on a whole-brain anatomical network or task-based functional networks and task performance in the reading task. **(A)** Correlation between reconfiguration efficiency in the task-based regional network and task behavior performance scores in the Bclp group. **(B)** Correlation between reconfiguration efficiency in the task-based regional network and task behavior performance scores in the Aclp group. **(C)** Correlation between reconfiguration efficiency based on the whole-brain anatomical network and task behavior performance scores in the Bclp group. **(D)** Correlation of reconfiguration efficiency based on the whole-brain anatomical network and task behavior performance scores in the Aclp group. Error lines in the figure are standard deviations (SDs).

We tested the correlations between reconfiguration efficiency for the reading task and behavioral performance in CLP patients. CLP patients’ behavioral performance was scored on the CSITWL. We found that the reconfiguration efficiency in CLP patients, whether based on a task-based regional network or whole-brain anatomical network, showed positive correlations with behavioral performance scores. As shown in Figure 6, the CLP patients with greater reconfiguration efficiency performed better in the reading task.

Taken together, these results demonstrate the impact of rehabilitation training on brain mechanisms in CLP patients. During the course of rehabilitation, brain function was restored in CLP patients, and their task performance and reconfiguration efficiency were closely related.

## 4 Discussion

How to evaluate the effect of rehabilitation on the improvement of brain function in CLP patients is a very important matter. Currently, the CSITWL scale is mainly used clinically to evaluate patients’ task performance, but it is not yet clear how the patients’ brain mechanisms are altered. To shed light on the foregoing, we scanned participants again during rest and during the task. Participants completed both reading and subvocalization tasks. Three main findings emerged. One was that Speech rehabilitation could improve language-related brain activation in CLP patients, and that patients who underwent rehabilitation had levels of brain activation for the task that were closer to those of normal controls. Next, the second was that reconfiguration efficiency in the whole-brain network better reflects rehabilitation effects than strongly correlated regional networks, and our study found that rehabilitated CLP patients had higher whole-brain reconfiguration efficiency than CLP patients without rehabilitation, which is consistent with their better reading performance. In addition, we also examined the effect of reaction time on reconfiguration efficiency and found that participants’ reconfiguration efficiency is independent of reaction time. Below, we will discuss these findings in more detail, providing information on how rehabilitation improves patients’ brain mechanisms and suggest future directions.

### 4.1 Speech rehabilitation could improve language-related brain activation in CLP patients

The flexibility to adapt one’s behavior to different environments is an essential human ability. Our sensory-to-motor mapping rules need to be constantly adapted to the current task requirements (Heinzle et al., 2012). Depending on the actual situation, the same sensory input may require two different motor responses. It has been proposed that FC within the cerebral cortex is biased to direct the flow of information according to the desired sensory-to-motor mapping (Neubauer and Fink, 2009; Lehne et al., 2014; Petersen and Sporns, 2015). We used fMRI to investigate whether rehabilitation truly changes motor responses in brain networks (Heeger and Ress, 2002; Logothetis, 2008; Craddock et al., 2012).

Regarding the reading task, in normal controls, brain areas such as the motor cortex and Broca’s area were activated. Additionally, there was strong activation in the cerebellum, Wernicke’s area, and occipital lobe, which is consistent with the activation results for Chinese semantic processing in other studies (Sato-Wakabayashi et al., 2008), suggesting that these brain regions are related to language comprehension (Rogalsky et al., 2011; Lim et al., 2018). Comparing the differential activation results between groups, our study found that the Bclp group had weaker intragroup activation intensity and a smaller activation range than the Norm group. The Aclp group showed essentially the same activation intensity in the motor cortex, cerebellum, fusiform gyrus, and insula as the Norm group. This result was attributed to a standardized rehabilitation model, showing that speech rehabilitation can restore brain functions related to speech and restore CLP patients’ brain functions to normal levels (Poldrack, 2015).

The Norm group showed that subvocalization produces similar activation results as reading. Comparing the differential activation between groups, the results showed that the Bclp group had a weaker intragroup activation intensity and a smaller activation range than the Norm and Aclp groups. However, the intensity of activation in the inferior frontal gyrus, fusiform gyrus, and cerebellum in the Aclp group was significantly different from that in the Norm group. This finding reflects the mechanism of rehabilitation, i.e., the purpose of rehabilitation is to restore only the functions related to speech. If one does not vocalize but simply mentally mumbles, it does not reflect well on the effectiveness of the rehabilitation.

### 4.2 Reconfiguration efficiency in the whole-brain network better reflects rehabilitation effects than strongly correlated regional networks

Brain network topology can offer essential information about healthy and pathological brain function (Cole et al., 2014). New approaches to brain network analysis have shown a link between topological properties and cognitive function (Mill et al., 2017). In previous studies, the exploration of brain features has typically focused on the connectivity patterns of the most strongly linked regions. Recent studies have shown that differences in intelligence quotient (IQ) levels are mainly due to the distributed communication efficiency of brain networks with more long-range moderate weak connections and less contribution from strong connections, and that changes in individual IQ are related to the overall efficiency of the brain rather than the efficiency strength of the relevant regions of the brain (Csermely, 2004; Santarnecchi et al., 2014). There is evidence that the optimization of information processing in the human brain depends on strong and weak connections, and that weak connections may be useful markers of specific pathological states (Bassett and Bullmore, 2009; Bassett et al., 2012).

We used two brain network templates to verify our conjectures. The first brain network template was based on task-specific locally activated brain areas, and we selected 12 ROIs for the reading task and 8 ROIs for the subvocalization task by intersecting the differentially activated brain areas in the three groups of participants across tasks with spheres of 5- or 8-mm radius and combined the selected ROIs into functional network atlases. We assessed the contribution of this network by separately calculating the task-based functional network FC reconfiguration efficiency for each participant. Afterward, we compared the correlation between reconfiguration efficiency and task performance in CLP patients. Our results showed that rehabilitation had a significant effect on task-based functional network reconfiguration efficiency. Significant differences in reconfiguration efficiency were found between the Bclp and Aclp groups in the reading task and between the Bclp and Aclp groups and between the Bclp and Norm groups in the subvocalization task. The second brain atlas was the AAL-1024 brain atlas based on the whole-brain anatomical network. In a similar manner, we calculated the reconfiguration efficiency in participants and the correlation between reconfiguration efficiency and behavioral performance. We found that the whole-brain-based anatomical network yielded some different results: in the reading task, there were significant differences between the Bclp and Aclp groups and between the Bclp and Norm groups; however, in the subvocalization task, there were no significant differences among participants in all three groups. Meanwhile, in the reading task, there was a strong positive correlation between the reconfiguration efficiency and the participants’ task performance. Our results suggested that when task-based regional networks are used to measure participants’ reconfiguration efficiency, the reconfiguration efficiency in those functional networks that are particularly important for a given task has a privileged effect on the performance in that task. In the present study, both the reading task-based brain atlas and the subvocalization task-based brain template were related to language function, and speech rehabilitation had a strong restorative effect on these language-related brain networks. When we considered the brain as a singular structure performing the task, the reconfiguration efficiency based on the whole-brain anatomical network showed that speech rehabilitation altered the reconfiguration efficiency in the reading task in CLP patients but had no significant improvement effect in the subvocalization task.

These results confirm our hypothesis. Reconfiguration efficiency in a locally strongly connected brain network does not reflect the true recovery of the patient. The brain works as a whole, using whole-brain-based reconfiguration efficiency is the only way to objectively reflect the ability of CLP patients to work in a coordinated manner while performing a specific task. Patients with CLP have functional deficits in articulation, but not in subvocalization, thinking, and other functions. We performed rehabilitation only for reading ability of CLP patients, and the results of whole-brain-based calculations showed that the CLP patients with rehabilitation had significantly higher brain reconfiguration efficiency in reading tasks, and similarly, there was no significant difference in the performance of the three groups in subvocalization tasks without specific training.

In addition, the efficiency of brain reconfiguration, as evaluated by whole-brain network analysis and fMRI, can provide more detailed information to guide personalized rehabilitation treatment in CLP patients. Unlike other traditional performance metrics, reconfiguration efficiency reveals how the brain adapts and integrates information across regions during tasks, enabling clinicians to target specific deficits. fMRI helps monitor this process in real time, allowing for adjustments in rehabilitation plans based on individual progress. For example, if certain language-related brain areas are under-activated, rehabilitation can be directed toward improving the connectivity in those areas. In this way, the rehabilitation strategy becomes dynamic and tailored to the individual for maximum effectiveness. Additionally, fMRI allows clinicians to track gains or no gains in specific brain networks, which ensures that interventions are optimized and relevant. This approach ultimately enhances speech rehabilitation with greater precision, offering a more effective and individualized approach for CLP patients.

### 4.3 Participants’ reconfiguration efficiency is independent of reaction time

The individuals who performed better in the reading task had more efficient whole-brain FC reconfiguration (Schultz and Cole, 2016; Hearne et al., 2017); nevertheless, participants who performed better typically had faster responses and took less time to complete the task (Poldrack, 2015). These individuals have more “rest time” while on assignment, which may lead to a higher similarity between task-based FC and resting-state FC for participants who performed better compared to those who perform relatively poorly on the task (Heitz, 2014). For this reason, we calculated the median reaction time for each participant and conducted a regression analysis of the reaction times based on our reconfiguration efficiency. Relating the post regression reconfiguration efficiency to participants’ CSITWL scores, we found that for the reading task, FC reconfiguration efficiency remained positively correlated with scale scores, indicating that reaction time did not affect our main findings.

Brain is a complex network in which spatially diverse information is constantly processed and transmitted between functionally connected regions (Cole et al., 2010; Zalesky et al., 2014). Recent studies have shown that FC in brain networks is organized in an efficient small-world format (Reid et al., 2019), suggesting that high levels of local neighborhood aggregation, as well as the presence of more distant connections, Ensure a high level of global communication efficiency across the network (Cole et al., 2010; Li et al., 2019). One study researched the overall structure of the brain network and suggested task ability is related to the efficiency of the organization of global connections in our brain and the efficiency of global information integration between different regions of the brain network, i.e., the reconfiguration efficiency used in this study (Cole et al., 2010; Zalesky et al., 2014; Schultz and Cole, 2016). Our findings suggest that we can use reconfiguration efficiency as an assessment index for speech intelligibility to evaluate the effectiveness of rehabilitation in the brains of CLP patients. Calculating the reconfiguration efficiency in CLP patients with rehabilitation can provide a clearer picture of the degree of brain function recovery in CLP patients.

## Data Availability

The original contributions presented in this study are included in this article, further inquiries can be directed to the corresponding authors.
